# The Effect of PM_2.5_ from Household Combustion on Life Expectancy in Sub-Saharan Africa

**DOI:** 10.3390/ijerph15040748

**Published:** 2018-04-13

**Authors:** Badamassi Aboubacar, Xu Deyi, Mahaman Yacoubou Abdoul Razak, Boubacar Hamidou Leyla

**Affiliations:** 1 School of Economics and Management, China University of Geosciences, Wuhan 430074, China; xdy@cug.edu.cn; 2 Department of Pathophysiology, School of Basic Medicine, Key Laboratory of Education Ministry of China for Neurological Disorders, Tongji Medical College, Huazhong University of Science and Technology, Wuhan 430030, China; razakmahaman@yahoo.fr; 3 Key Laboratory of Tectonics and Petroleum Resources of Ministry of Education, China University of Geosciences, Wuhan 430074, China; leyla_boubacar@yahoo.fr

**Keywords:** household combustion, PM_2.5_, life expectancy (LEX), biomass fuel, solid fuel, general method of moments (GMM), panel cointegration, Sub-Saharan Africa (SSA)

## Abstract

Household fuel combustion, especially using solid combustibles (biomass and fossil fuels), for cooking and other activities produces emissions that contribute to concentrations of indoor as well as outdoor air pollutants such as particulate matter with diameter smaller than 2.5 μm (PM_2.5_) that deteriorate health and likely affect life expectancy (LEX). This study investigates the impact of PM_2.5_ from household combustion on LEX considering several covariates while controlling for ambient PM_2.5_ generated by other sectors. The generalized method of moments (GMM) model and the panel cointegration model were applied to a dataset of 43 Sub-Saharan Africa (SSA) countries over the time period of 1995–2010. Both approaches provide similar results indicating that household PM_2.5_ is significantly and negatively associated with higher aggregate LEX in the long-run, and, to a greater degree for female’s. Also, among the control variables, PM_2.5_ from the transport sector has a greater influence on male’s LEX. Thus, efforts should be combined to reduce household PM_2.5_ since lower levels are associated with increased LEX.

## 1. Introduction

Heating, lighting, cooking and other activities, especially with solid fuels in the household (residential or domestic) sector, cause the emission of air pollutants including particulate matter with diameter smaller than 2.5 μm (PM_2.5_), causing indoor and outdoor air pollution [1,2,3,4,5,6]. Despite progress in the energy sector, household combustion in Sub-Saharan Africa (SSA), as in many other developing countries, and especially in the rural areas, still, predominantly involves the use of solid fuels for cooking and other domestic energy requirements [7], mainly due to the poor economic conditions of households and lack of access to cleaner fuels [8]. Moreover, more than 3 billion people globally rely on the combustion of solid fuels for cooking [9], of which 700 million are Africans [10].

Emissions from household combustion greatly contribute to the concentrations of toxic air pollutants that are harmful to human health. Exposure to concentrations of air pollutants from household emissions is higher indoors, and more greatly affects women and children who spend more time in the household [11]. There is a large body of evidence indicating that exposure to air pollution from household combustion emissions, especially fine particulate matter air pollution (PM_2.5_) has damaging impacts on pulmonary and cardiovascular health [12,13,14,15] and causes child pneumonia [16]. These damaging health impacts are observed in increased mortality risks [17,18,19,20] and are also reflected in changes in human life expectancy, as recently suggested [21,22,23,24,25,26,27,28].

However, despite the well documented literature on the association between PM_2.5_ and health problems, studies regarding the effects of PM_2.5_ on life expectancy are still globally scarce [24,29,30,31,32], particularly those covering Sub-Saharan African countries where the precise case of household air pollution from household combustion using solid combustibles (biomass and fossil fuels) is a major public health and environmental concern, as is the lower general human life expectancy in this area as compared to the other regions of world.

Therefore, the purpose of this study is to briefly examine the role of PM_2.5_ from household combustion in determining life expectancy in Sub-Saharan Africa while using several covariates and controlling for ambient PM_2.5_ generated by combustion from other sources. We employ empirical approaches because literature regarding the nexus between household combustion PM_2.5_ and its health-related adverse effects in Sub-Saharan Africa is scarce.

## 2. Methods

### 2.1. Data

**Figure 1:**
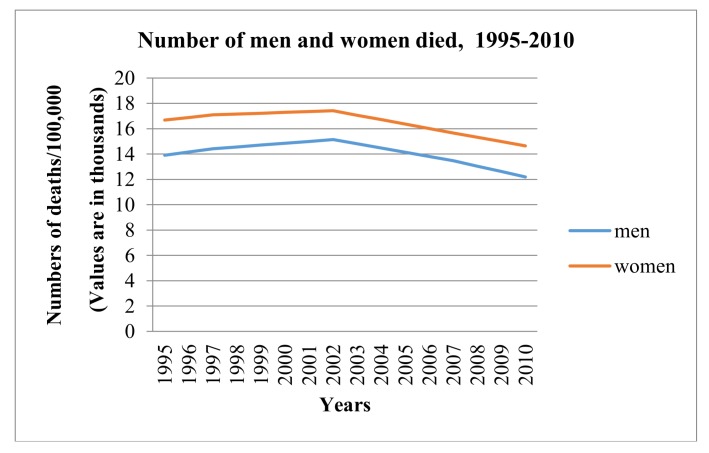
Number of deaths (men and women), 1995–2010.

The study population consisted of 597,024,836 people, of which 300,126,872 were women and 296,897,964 were men. We assembled a dataset of 43 sub-Saharan countries over the time period 1995–2010, with annual country PM_2.5_ from household combustion, aggregate life expectancy (ALEX), female life expectancy (FLEX) and male life expectancy (MLEX) considered as the main variables. We also used ambient PM_2.5_ data on combustion from other sectors (categorized into transportation, manufacturing industries and construction, and other sectors) as control variables. The covariate variables are: gross domestic product (GDP) per capita growth rate, health expenditure per capita (HEXP), prevalence of HIV/AIDS in the population (P-HIV/AIDS), prevalence of undernourished people in the population (PUNP), proportion of urban population in the country (PUP), and proportion of population with primary school education (PPS). This dataset was formed based on the availability of data and all data were on yearly basis. The total emissions of PM_2.5_ from 1995 to 2010 in this area were 5.16 × 10^4^ Gg, and the proportion of the main source (household combustion) was 91.25%. Moreover, Figure 1 shows the number of men and women who died between 1995 and 2010 in the study population; from the figure it can be seen that there has been a decreasing trend in mortality since 2002. Health expenditure data and PM_2.5_ data were transformed into logarithmic form before running the regressions as they were not expressed in percentages while the other explanatory variables were to better interpret the results.

The PM_2.5_ data were sourced from the database Emissions Database for Global Atmospheric Research version v4.3.1 (EDGARv4.3.1) of the European Commission of the European Union. The latest release was used when writing this paper; and all the remaining data on covariates were taken from the world development indicators (WDI) of the World Bank.

We used GDP in order to control for the wealth of countries because greater income can affect public health through better access to health care, improving wellbeing and standards of living [33,34]. The HEXP is considered since the more countries invest in health expenditure, the more public health is likely to improve. The P-HIV/AIDS and PUNP are used because of their importance in determining life expectancy, especially in SSA where they are serious public health issues. PU is used since more urbanized countries are more likely to provide better health care and improve public health.

### 2.2. Models

For the empirical analyses, this study used two (2) different models: the panel cointegration regression model and the two-step system generalized method of moments (GMM) model. These two different approaches are used in order to compare the results and assess their robustness. Below are details regarding the models.

#### 2.2.1. Two-Step System Generalized Method of Moments (GMM) Model

The model is expressed as follows:

(1) $$Y_{it}=\overset{h}{\underset{f=1}{\sum }}\beta_{1}Y_{it-f}+\gamma_{l}X_{it-l}+\delta_{i}\;+\;\varepsilon_{it}$$

(2) $$E\left[\delta_{i}\right]=E\left[\varepsilon_{it}\right]=E\left[\delta_{i}\varepsilon_{it}\right]=0$$

where *Y* represents the dependent variables, which are the three life expectancy (aggregate, female, male) variables namely ALEX, FLEX, and MLEX of country *i* in year *t*; *Y_it−f_* denotes *Y* previous (lagged or past or delayed) years’ values. This means that the present year’s life expectancy depends also on the previous years’ life expectancies, or in other words, future years’ life expectancies depend on the past years’ life expectancies. Hence, this dependency shows that the effect of PM_2.5_ does not immediately lead to human death, there is a delay). *X* stands for the vector of all the independent variables (where PM_2.5_ and HEXP variables are in logarithmic form) of country *i* in year *t*; $\delta_{i}$ refers to the country local-specific characteristics (for example, the fact that households use of fuel types is correlated with local energy distribution); and $\varepsilon_{it}$ is the observations error term; while $E\left[\delta_{i}\right]=E[\varepsilon_{it}]=E[\delta_{i}\varepsilon_{it}]=0$ means that $\delta_{i}$ and $\varepsilon_{it}$ are uncorrelated.

There are several reasons for choosing GMM; to control endogeneity problems between explanatory variables (for example GDP per capita and the household combustion PM_2.5_ variable might be endogenous because the use of fuel type by households might be correlated with the economic situation of the household); to considere country local-specific characteristics (for example, the fact that household use of fuel types is correlated with local energy distribution); to deal with many countries and a shorter time period; to remove the time-invariant characteristics of countries (time-invariant country heterogeneity like geography could be correlated with fuel use—for example, one might think that people of Sub-Saharan African countries located in more forested areas would be more likely to use wood and charcoal as fuels in their households); and to control for autocorrelation (that may rise due to the presence of the lagged (delayed) effect of dependent variable).

All the empirical analyses were carried out with the software STATA (version 14, Stata Corporation, College Station, TX, USA).

#### 2.2.2. Panel Cointegration Regression Model

The model is specified as follows:

(3) $$Y_{it\;}=\;\beta_{i}+\;\overset{10}{\underset{n=1}{\sum }}\alpha_{ni}X_{it}+\;\varepsilon_{it}\;$$

where *Y_it_* represents the dependent variables, which are the three life expectancy (aggregate, female, male) variables, namely the ALEX, FLEX, and MLEX of country *i* in year *t*; *X* stands for the vector of all the independent variables (where PM_2.5_ and HEXP variables are in a logarithmic form) of country *i* in year *t*; $\beta_{i}$ refers to country local-specific characteristics (for example, the fact that the use of fuel types is correlated with local energy distribution); $\alpha_{ni}$ indicates the country coefficients related to the n independent variables; and $\varepsilon_{it}$ is the observations error term.

An advantage of employing this model is that it enables an investigation of whether PM_2.5_ from household combustion has a long-run effect (effect with delay) or a short-run effect (immediate effect) on the three different life expectancy (aggregate, female, male) variables. However, following the standard procedure, before estimating the long-run and short-run parameters, some properties of the variables have to be investigated using the following tests:

1. Panel unit root tests: to check whether the variables are stationary or not at level; or whether they are stationary at their first difference (integrated of order one);
2. Panel cointegration tests: to check whether there exists a long-run relationship between the variables.

Other advantages of using this model are that: it considers country local-specific characteristics (for example, the fact that household use of fuel types is correlated with local energy distribution), it controls for endogeneity (for example GDP per capita and the household combustion PM_2.5_ variable might be endogenous because the use of fuel types by households might be correlated with the economic situation of the household) and omitted variable problems.

## 3. Results

### 3.1. Results of the Two-Step System Generalized Method of Moments (GMM) Model

We estimate three separate regressions, each considering a life expectancy variable (aggregate, female, male) as the dependent variable. However, before running the three separate regressions, we conducted a Pearson correlation analysis to test whether some of the explanatory variables are correlated and found that there is no significant correlation between the variables; the results are not reported here for the sake of saving space but are available upon request. Additionally, after the regressions, the Hansen-J test, and the Arellano–Bond test were carried out to check the validity of the estimated results; and they were revealed to be good. More details regarding the reasons and the procedure for choosing this technique as well as the validity tests are as previously reported in [35,36,37].

Table 1 presents the results of the three estimated regressions for the impact of domestic combustion PM_2.5_ on the three different life expectancy (LEX) variables (aggregate life expectancy (ALEX), female life expectancy (FLEX), and male life expectancy (MLEX)) while controlling the PM_2.5_ resulting from combustion by other sectors and other covariates. There is a greater focus on the discussion of the main interest variables.

Since the Hansen-J test and the Arellano–Bond (AR) (2) tests—which have the following respective null hypotheses: “the instruments as a group are exogenous” and “there is no autocorrelation”—are all accepted (because *p*-values of 0.321, 0.186, 0.218 and 0.427, 0.266, 0.489 are all greater than 0.05), the estimated results are all valid.

**Figure 2:**
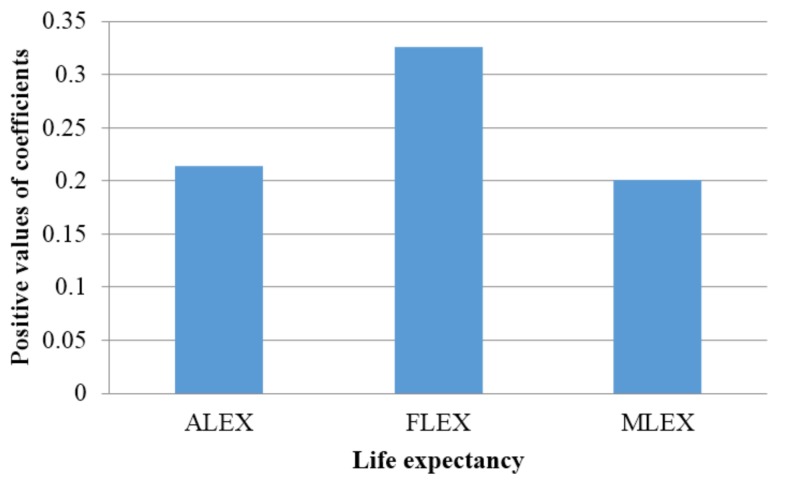
Comparison of magnitudes of impact of PM_2.5H_ on the three variables of life expectancy. Note: ALEX indicates aggregate life expectancy; FLEX is female life expectancy; MLEX refers to male life expectancy.

It can be seen that PM_2.5_ from household combustion is significantly and negatively associated with greater life expectancy effects across all the three regressions but with higher impact on female life expectancy (FLEX) (Table 1 and Figure 2). These findings suggest that an increase in PM_2.5_ from household combustion is associated with a decrease in life expectancy; more precisely, an increase of 10% in household combustion PM_2.5_ is associated with a decrease of 0.214 years, 0.326 years, and 0.201 years in ALEX, FLEX, and MLEX respectively, holding other factors constant. Meanwhile, PM_2.5_ from the transportation sector is also significantly and negatively related to the three life expectancy variables, but more greater affect male life expectancy (MLEX); thus when PM_2.5_ from transportation goes up by 10%, ALEX, FLEX, and MLEX values go down by 0.127 years, 0.095 years, and 0.182 years respectively, ceteris paribus. Regarding other covariates, only the PM_2.5_ from manufacturing industries and construction, PM_2.5_ from other sectors, and the proportion of population with primary school education (PPS) are insignificant. GDP per capita growth rate, health expenditure per capita (HEXP) and proportion of urban population in country (PUP) all positively affect the three life expectancy variables, indicating that an increase in these three covariates is associated with an increase in the three life expectancy variables. The coefficients of GDP with respect to the three life expectancy variables are 0.022, 0.028, and 0.017, ceteris paribus. Moreover, the coefficients of HEXP are 0.038, 0.044, and 0.029 whereas those of PUP are 0.021, 0.016, and 0.024, ceteris paribus. Furthermore, prevalence of HIV/AIDS in population (P-HIV/AIDS) and prevalence of undernourished people in the population (PUNP) are both significantly and negatively correlated with the three life expectancy variables suggesting that an increase in both independent variables is associated with a decrease in the three life expectancy variables. While the coefficients of P-HIV/AIDS are −0.206, −0.183, and −0.213, those of PUNP are −0.067, −0.045, and −0.038, holding other factors constant.

### 3.2. Results of Panel Cointegration Regression Model

#### 3.2.1. Panel Unit Root Tests

As is necessary, the first step is to check the integrational properties of the variables, precisely whether the series (variables) are stationary or not at level. In the case they are non-stationary, their first difference series should be before proceeding to the next step (cointegration). In order to achieve this objective and assess the robustness of the results, we used two different panel unit root tests, precisely the Im, Pesaran and Shin (IPS) panel unit root test [38] and the Breitung panel unit root test [39]. The results are reported in Table 2. Both tests accept the null hypothesis of non-stationary variables at level (since their *p*-values are greater than 0.01 (1%) or 0.05 (5%)); but they could not fail to reject it after the first difference of variables (as their *p*-values are less than 0.01 (1%) or 0.05 (5%)); meaning that all the series are stationary at the first order (or are integrated of order one). This paved the way for panel cointegration analysis, an important step in distinguishing between long-run and short-run impacts.

#### 3.2.2. Panel Cointegration Tests

The next step is to test for cointegration since we previously found first-order stationary variables. Thus, we used the [40] panel cointegration test which presented seven (7) different panel test statistics, as reported in Table 3. All the tests rejected the null hypothesis of no cointegration (since their *p*-values are less than 0.01 (1%) or 0.05 (5%)), suggesting that the variables are indeed cointegrated, hence meaning that there exists a long-run relationship among variables.

#### 3.2.3. Estimates of the Long-Run and Short-Run effects

The long-run effects of PM_2.5_ and other covariates on life expectancy are generated using panel ordinary least square (OLS) and panel dynamic ordinary least square (DOLS) estimators. The use of both estimators is in order to assess the robustness of the results. The estimates are reported in Table 4. The results from both estimators provided similar values of the long-run coefficients which are also similar to the estimates provided by the GMM model.

Then, estimates of the short-run effects are obtained using the first difference variables; and by applying panel error correction method where the error correction term (ECT) is computed from the long-run cointegrating relationship. The results are reported in Table 5.

Starting with the long-run results, our main findings indicate that all the variables significantly affect life expectancy in the long-run. More importantly, the coefficient of the household’s PM_2.5_ is negative and with a higher impact on female life expectancy (FLEX), holding other factors constant. For instance, the findings suggest that a 10% increase in PM_2.5H_ decreases aggregate, male and female life expectancy by around 0.216 years, 0.324 years and 0.206 years, respectively, in the long-run. Further, PM_2.5_ from transportation sector also impacts significantly and negatively the three life expectancy variables with a bigger magnitude with respect to male life expectancy (MLEX); hence when PM_2.5_ from transportation increases by 10%, ALEX, FLEX and MLEX values are reduced by approximately 0.129 years, 0.095 years and 0.181 years, respectively, in the long-run. Concerning other covariates, only PM_2.5_ from manufacturing industries and construction, PM_2.5_ from other sectors and the proportion of population with primary school education (PPS) are insignificant. The long-run effects of GDP per capita growth rate (GDP), health expenditure per capita (HEXP) and proportion of urban population in country (PUP) on the three life expectancy variables are all positive, suggesting that an increase in these three covariates is associated with an increase in the three life expectancy variables in the long-run. Thus, the long-run coefficients of GDP with respect to the three life expectancy variables are approximately 0.022, 0.027, and 0.019; those of HEXP are approximately 0.037, 0.043, and 0.029, and those of PUP are approximately 0.022, 0.014, and 0.025, ceteris paribus. Moreover, prevalence of HIV/AIDS in population (P-HIV/AIDS) and prevalence of undernourished people in population (PUNP) are both significantly and negatively related with the three life expectancy variables in the long-run, indicating that an increase in both independent variables is associated with a decrease in the three life expectancy variables. The long-run coefficients of P-HIV/AIDS are approximately −0.205, −0.184, and −0.211, while those of PUNP are approximately −0.068, −0.045, and −0.038, holding other factors constant.

Now, turning to the short-run results, the findings indicate that only GDP and HEXP significantly affect the three life expectancy variables in the short-run (as reflected by the coefficients of the terms ΔGDP_t−1_ and Δln HEXP_t−1_). More interestingly PM_2.5H_ does not significantly affect the three life expectancy variables in the short-run.

In general, the findings reveal more importantly that PM_2.5_ from household combustion (PM_2.5H_) does not have a significant short-run impact on the three life expectancy variables but it has a long-run effect on them.

## 4. Discussion

This study investigated the role of PM_2.5_ from household combustion in determining life expectancy in Sub-Saharan Africa. We distinguished our study from previous works by employing two (2) different approaches (generalized method of moments (GMM) model and the panel cointegration model) which provide similar and robust results. The findings suggest that, in addition to PM_2.5_ from household combustion, several other factors affect life expectancy in the region with some of them having negative impacts and others positive effects. Interestingly, except for GDP and health expenditure variables that have short-run and long-run impacts on life expectancy, PM_2.5_ from household combustion and all other covariates affect life expectancy only in the long-run. More importantly, an increase of PM_2.5_ from household combustion is associated with a decrease in life expectancy; and the magnitude of the effect is higher for female life expectancy. Studies [25,41] have shown similar results using cross-sectional data techniques. An explanation for this could be the fact that women in Sub-Saharan Africa are more exposed as compared to men since they spend much more time within household involved in cooking and other domestic activities. Another interesting finding is the significant and positive coefficient of proportion of urban population. Some possible reasons for this include the fact that people living in urban areas have higher standards of healthcare compared to rural populations, with lower disease incidence and different health behaviors. Further, an additional important aspect of this study is the positive significance of GDP per capita growth rate, which suggest that a gain in life expectancy could be achieved through an improvement in economic conditions in Sub-Saharan Africa; implying that, with better economic situations, households are more likely to use cleaner fuels, implying a decrease in large PM_2.5_ from household combustion, ceteris paribus. Along the same line, previous studies [22,23,24,25,26,27,28,41,42] found evidence that reduction in air pollution is associated with increase in changes in life expectancy. Moreover, the findings of this study also have the following implications: household combustion PM_2.5_ is associated with life expectancy; solid fuel combustion highly dominates domestic combustion of fuels in Sub-Saharan Africa; and the within-household exposure to concentrations of air pollutants from domestic combustion emissions is high; hence, by extending these analyses, there is a possible association between exposure to other household air pollutants from household combustion emissions and life expectancy in Sub-Saharan Africa.

A novel aspect of this study is that we extend the existing literature by establishing empirical relationships between PM_2.5_ from household combustion and life expectancy in Sub-Saharan Africa using methods that simultaneously capture both long-run and short-run effects. This study could also contribute importantly to public health and air pollution management in the region.

However, this study has some limitations. First, it uses country-level data while household-level data could better capture the relationships between household PM_2.5_ and life expectancy in relation to several socio-economic, demographic and other factors in Sub-Saharan Africa; this is due to the unavailability of household-level data. Additionally, several other factors such as smoking prevalence could also affect life expectancy, but because of lack of data we did not use them. Thus, care should be taken when interpreting the findings of this study; further research should be conducted using more advanced methods.

Mainly, the findings of this study suggest that PM_2.5_ from household combustion is negatively associated with life expectancy and with greater effect on female life expectancy; thus, private and public policy efforts should be combined to reduce PM_2.5_ in the household combustion sector.

## 5. Conclusions

This study examined the effect of household combustion PM_2.5_ on life expectancy in Sub-Saharan Africa. The main findings revealed that PM_2.5_ from household combustion is negatively associated with life expectancy in the long-run and with a greater effect on female’s life expectancy. Hence, these results suggest that efforts should be combined in order to reduce household combustion PM_2.5_ as its decrease is associated with increase in life expectancy. However, despite its interesting findings, this study has some limitations that could be further investigated.

## Figures and Tables

**Table 1:** Estimated regression coefficients for the effect of particulate matter with diameter smaller than 2.5 µm (PM_2.5_) from household combustion and other variables on the three (3) life expectancy variables.

Variables	ALEX	FLEX	MLEX
ALEX(−1)	−0.791 * (0.084)		
FLEX(−1)		−0.835 * (0.056)	
MLEX(−1)			−0.773 *(0.079)
ln (PM_2.5H_) × 10	−0.214 *** (0.007)	−0.326 *** (0.000)	−0.201 ** (0.015)
ln (PM_2.5TR_) × 10	−0.127 ** (0.036)	−0.095 ** (0.042)	−0.182 ** (0.021)
ln (PM_2.5MC_) × 10	−0.921 (0.584)	−0.110 (0.192)	−0.139 (0.403)
ln (PM_2.5OT_) × 10	−0.083 (0.617)	−0.065 (0.235)	−0.086 (0.148)
GDP	0.022 ** (0.011)	0.028 *** (0.001)	0.017 ** (0.029)
ln HEXP	0.038 *** (0.003)	0.044 *** (0.001)	0.029 *** (0.008)
P-HIV/AIDS	−0.206 *** (0.000)	−0.183 *** (0.000)	−0.213 *** (0.004)
PUNP	−0.067 ** (0.031)	−0.045 ** (0.024)	−0.038 *** (0.001)
PUP	0.021 ** (0.025)	0.016 ** (0.014)	0.024 ** (0.047)
PPS	0.001 (0.304)	0.009 (0.712)	0.013 (0.459)
Validity Tests			
Countries	43	43	43
AR(1) (*p*-value)	0.008	0.029	0.041
AR(2) (*p*-value)	0.427	0.266	0.489
Hansen-J test (*p*-value)	0.321	0.186	0.218

Note: ALEX indicates aggregate life expectancy; FLEX is female life expectancy; MLEX refers to male life expectancy; ALEX(−1) refers to the lagged previous period of aggregate life expectancy; FLEX(−1) refers to the lagged previous period of female life expectancy; MLEX(−1) refers to the lagged previous period of male life expectancy; ln (PM_2.5H_) stands for the logarithm of PM_2.5_ from household combustion; ln (PM_2.5TR_) stands for the logarithm of PM_2.5_ from the transportation sector; ln (PM_2.5MC_) stands for the logarithm of PM_2.5_ from manufacturing industries and construction; ln (PM_2.5OT_) stands for the logarithm of PM_2.5_ from other sectors; *p*-values are in parentheses; *** significant at 1%; ** significant at 5%; * significant at 10%; GMM stands for generalized method of moments; AR( ) refers to the Arellano–Bond test; GDP refers to gross domestic product; HEXP refers to the health expenditure per capita; ln HEXP refers to the logarithm of health expenditure per capita; P-HIV/AIDS refers to the prevalence of HIV/AIDS in the population; PUNP refers to the prevalence of undernourished people in the population; PUP refers to the proportion of urban population in the country PPS refers to the proportion of population with primary school education.

**Table 2:** Results of panel unit root tests.

Variables	Breitungt-Test	IPS Test
ALEX	0.488 (0.211)	0.081 (0.365)
ΔALEX	−3.047 ** (0.027)	−5.131 *** (0.001)
FLEX	0.269 (0.638)	0.106 (0.411)
ΔFLEX	−1.935 *** (0.000)	−6.064 *** (0.005)
MLEX	2.071 (0.519)	−0.822 (0.396)
ΔMLEX	0.759 ** (0.012)	0.741 ** (0.036)
ln PM_2.5H_	0.0258 (0.417)	−0.8233 (0.862)
Δln PM_2.5H_	−2.380 ** (0.026)	−4.751 *** (0.000)
ln PM_2.5TR_	0.609 (0.473)	0.0185 (0.781)
Δln PM_2.5TR_	−3.774 *** (0.008)	−5.136 ** (0.042)
ln PM_2.5MC_	−1.021 (0.849)	2.104 (0.137)
Δln PM_2.5MC_	−4.265 *** (0.002)	−4.791 *** (0.000)
ln PM_2.5OT_	0.936 (0.473)	1.480 (0.805)
Δln PM_2.5OT_	−1.294 *** (0.009)	−3.151 ** (0.042)
GDP	2.811 (0.863)	−0.602 (0.158)
ΔGDP	−2.507 ** (0.019)	−8.446 ** (0.025)
ln HEXP	0.543 (0.618)	3.104 (0.107)
Δln HEXP	−5.192 *** (0.001)	−6.425 *** (0.000)
P-HIV/AIDS	1.806 (0.274)	0.923 (0.405)
ΔP-HIV/AIDS	−3.188 ** (0.031)	−4.209 *** (0.000)
PUNP	0.529 (0.148)	0.174 (0.362)
ΔPUNP	−0.328 ** (0.027)	−1.566 ** (0.018)
PUP	6.106 (0.593)	2.078 (0.494)
ΔPUP	−1.355 *** (0.006)	−3.921 *** (0.002)
PPS	2.602 (0.346)	0.917 (0.496)
ΔPPS	−4.180 ** (0.040)	−5.621 ** (0.029)

Note: *p*-values are in parentheses and **; *** denote statistical significance at the 5% and 1% levels, respectively; Δvariable indicates first difference of the variable; Δln variable indicates first difference of the logarithm of the variable; PM_2.5H_ is PM_2.5_ from household combustion; PM_2.5TR_ is PM_2.5_ from transport; PM_2.5MC_ is PM_2.5_ from manufacturing industries and construction; PM_2.5OT_ is PM_2.5_ from other sectors; and IPS refers to the Im, Pesaran and Shin panel root test.

**Table 3:** Results of the panel cointegration test for aggregate life expectancy (ALEX), female life expectancy (FLEX) and male life expectancy (MLEX).

Pedroni’s Test Statistics	ALEX	FLEX	MLEX
Panel v-statistics	−0.953 ** (0.038)	−4.0618 *** (0.003)	−2.373 *** (0.000)
Panel rho-statistics	−3.420 ** (0.014)	−1.171 ** (0.028)	−2.851 *** (0.000)
Panel pp-statistics	−2.337 *** (0.000)	−0.853 ** (0.016)	−0.522 ** (0.036)
Panel ADF-statistics	−1.592 ** (0.023)	−0.649 *** (0.000)	−3.116 ** (0.017)
Group rho-statistics	−1.368 *** (0.001)	−1.796 *** (0.006)	−1.527 *** (0.009)
Group pp-statistics	−3.205 *** (0.002)	−2.057 ** (0.046)	−1.384 ** (0.025)
Group ADF-statistics	−2.141 *** (0.008)	−2.005 ** (0.023)	−3.469 ** (0.011)

Note: *p*-values are in parenthesis and **; *** denote statistical significance at the 5% and 1%, respectively.

**Table 4:** Estimates of long-run coefficients for aggregate life expectancy (ALEX), female life expectancy (FLEX) and male life expectancy (MLEX).

Long-Run Coefficients						
Variables	ALEX		FLEX		MLEX	
	Panel OLS	Panel DOLS	Panel OLS	Panel DOLS	Panel OLS	Panel DOLS
ln (PM_2.5H_) × 10	−0.216 *** (0.001)	−0.218 *** (0.000)	−0.324 *** (0.004)	−0.323 *** (0.009)	−0.206 *** (0.000)	−0.204 ** (0.023)
ln (PM_2.5TR_) × 10	−0.128 *** (0.008)	−0.129 ** (0.034)	−0.097 ** (0.021)	−0.095 ** (0.027)	−0.178 ** (0.044)	−0.181 ** (0.016)
ln (PM_2.5MC_) × 10	−0.901 (0.306)	−1.004 (0.850)	−0.108 (0.763)	−0.114 (0.333)	−0.133 (0.995)	−0.142 (0.617)
ln (PM_2.5OT_) × 10	−0.095 (0.621)	−0.089 (0.144)	−0.061 (0.291)	−0.069 (0.804)	−0.083 (0.104)	−0.087 (0.571)
GDP	0.023 ** (0.015)	0.022 ** (0.041)	0.026 ** (0.035)	0.027 ** (0.018)	0.018 *** (0.009)	0.019 *** (0.000)
ln HEXP	0.037 ** (0.027)	0.037 ** (0.011)	0.048 *** (0.000)	0.043 *** (0.003)	0.027 *** (0.001)	0.029 ** (0.012)
P-HIV/AIDS	−0.210 ** (0.033)	−0.205 *** (0.004)	−0.187 *** (0.001)	−0.184 *** (0.002)	−0.209 *** (0.007)	−0.211 *** (0.000)
PUNP	−0.067 ** (0.019)	−0.068 ** (0.025)	−0.043 ** (0.016)	−0.045 *** (0.000)	−0.037 ** (0.021)	−0.038 ** (0.017)
PUP	0.020 ** (0.043)	0.022 ** (0.012)	0.013 ** (0.039)	0.014 ** (0.022)	0.024 ** (0.011)	0.025 ** (0.038)
PPS	0.008 (0.172)	0.004 (0.532)	0.011 (0.466)	0.006 (0.395)	0.018 (0.275)	0.017 (0.611)

Note: *p*-values are in parentheses and **; *** denote statistical significance at the 5% and 1%, respectively; PM_2.5H_ is PM_2.5_ from household combustion; PM_2.5TR_ is PM_2.5_ from transport; PM_2.5MC_ is PM_2.5_ from manufacturing industries and construction; and PM_2.5OT_ is PM_2.5_ from other sectors; OLS refers to ordinary least square; DOLS refers to dynamic ordinary least square.

**Table 5:** Estimates of short-run coefficients for aggregate life expectancy (ALEX).

Short-Run Coefficients			
	ALEX	FLEX	MLEX
Δln (PM_2.5H_) × 10	−0.037 (0.659)	−0.029 (0.147)	−0.036 (0.638)
Δln (PM_2.5TR_) × 10	−0.015 (0.276)	−0.020 (0.821)	−0.014 (0.253)
Δln (PM_2.5MC_) × 10	0.125 (0.360)	0.153 (0.268)	0.205 (0.406)
Δln (PM_2.5OT_) × 10	−0.058 (0.204)	−0.049 (0.610)	−0.073 (0.120)
ΔGDP	0.054 (0.192)	0.083 (0.654)	0.061 (0.149)
Δln HEXP	0.046 (0.781)	0.137 (0.115)	0.183 (0.404)
ΔP-HIV/AIDS	0.108 (0.429)	0.216 (0.233)	0.195 (0.723)
ΔPUNP	0.021 (0.953)	0.018 (0.379)	0.024 (0.152)
ΔPUP	0.009 (0.357)	0.015 (0.162)	0.028 (0.337)
ΔPPS	0.037 (0.144)	0.061 (0.728)	0.049 (0.506)
ΔGDP_t−1_	0.019 ** (0.016)	0.021 ** (0.027)	0.012 ** (0.040)
Δln HEXP_t−1_	0.026 *** (0.000)	0.034 ** (0.013)	0.023 ** (0.037)
ECT_t−1_	−0.118 ** (0.039)	−0.126 ** (0.018)	−0.112 ** (0.045)

Note: *p*-values are in parenthesis and **; *** denote statistical significance at the 5% and 1%, respectively; Δ indicates first difference of the variable; PM_2.5H_ is PM_2.5_ from household combustion; PM_2.5TR_ is PM_2.5_ from transport; PM_2.5MC_ is PM_2.5_ from manufacturing industries and construction; PM_2.5OT_ is PM_2.5_ from other sectors and ECT_t−1_ refers to lagged previous period of error correction term.

## Abbreviations

PM_2.5_	Particulate Matter with diameter smaller than 2.5 μm
LEX	Life Expectancy
ALEX	Aggregate Life Expectancy
FLEX	Female Life Expectancy
MLEX	Male Life Expectancy
GDP	Gross Domestic Product
HEXP	Health Expenditure Per Capita
P-HIV/AIDS	Prevalence of HIV/AIDS
PUNP	Prevalence of Undernourished People
PUP	Proportion of Urban Population In Country
PPS	Proportion of Population with Primary School Education
GMM	Generalized Method of Moments
SSA	Sub-Saharan Africa
OLS	Ordinary Least Square
DOLS	Dynamic Ordinary Least Square
ECT	Error Correction Term
